# Multidisciplinary Smartphone-Based Interventions to Empower Patients With Acute Coronary Syndromes: Qualitative Study on Health Care Providers’ Perspectives

**DOI:** 10.2196/10183

**Published:** 2018-10-31

**Authors:** Nazli Bashi, Hamed Hassanzadeh, Marlien Varnfield, Yong Wee, Darren Walters, Mohanraj Karunanithi

**Affiliations:** 1 Australian e-Health Research Centre Herston Australia; 2 School of Medicine The University of Queensland Brisbane Australia; 3 Department of Cardiology Queensland Health Brisbane Australia

**Keywords:** acute coronary syndrome, focus group, health care professionals, mobile phone, multidisciplinary, thematic analysis

## Abstract

**Background:**

Postdischarge interventions are limited in patients with acute coronary syndrome (ACS) due to few scheduled visits to outpatient clinics and travel from remote areas. Smartphones have become a viable lifestyle technology to deliver educational and health interventions following discharge from hospital.

**Objective:**

The purpose of this study was to identify the requirements for the delivery of a mobile health intervention for the postdischarge management of patients with ACS via a multidisciplinary focus group.

**Methods:**

We conducted a focus group among health care professionals (n=10) from a large metropolitan hospital in May 2017. These participants from a multidisciplinary team contributed to a 1-hour discussion by responding to 8 questions relating to the applicability of smartphone-based educational and health interventions. Descriptive statistics of the focus group data were analyzed using SPSS. The qualitative data were analyzed according to relevant themes extracted from the focus group transcription, using a qualitative description software program (NVivo 11) and an ontology-based concept mapping approach.

**Results:**

The mean age of the participants was 47 (SD 8) years: 3 cardiologists; 2 nurse practitioners; 2 clinical nurses; 2 research scientists; and 1 physiotherapist. Of these participants, 70% (7/10) had experience using electronic health intervention during their professional practice. A total of 7 major themes and their subthemes emerged from the qualitative analysis. Health care providers indicated that comprehensive education on diet, particularly providing daily meal plans, is critical for patients with ACS. In terms of ACS symptoms, a strong recommendation was to focus on educating patients instead of daily monitoring of chest pain and shortness of breathing due to subjectivity and insufficient information for clinicians. Participants pointed that monitoring health measures such as blood pressure and body weight may result in increased awareness of patient physical health, yet may not be sufficient to support patients with ACS via the smartphone-based intervention. Therefore, monitoring pain and emotional status along with other health measures was recommended. Real-time support via FaceTime or video conferencing was indicated as motivational and supportive for patient engagement and self-monitoring. The general demographics of patients with ACS being older, having a low educational level, and a lack of computer skills were identified as potential barriers for engagement with the smartphone-based intervention.

**Conclusions:**

A smartphone-based program that incorporates the identified educational materials and health interventions would motivate patients with ACS to engage in the multidisciplinary intervention and improve their health outcomes following discharge from hospital.

## Introduction

Information and communication technologies are changing the form and quality of the delivery of health-related services, commonly known as electronic health (eHealth). As an emerging field in the intersection of medical informatics, public health, and business, eHealth refers to health services and information delivered or enhanced through internet-based technologies [1]. Mobile health (mHealth) is the subset of eHealth that refers to the delivery of health-related services via mobile communications technology. The examples of mHealth solutions include patient-provider communication, point-of-care data exchange, remote monitoring of medical devices, public health alerts, patient education, and clinical trials information [1].

mHealth applications play a significant role in shaping the future of the health care delivery system and have captured the attention of health care stakeholders. mHealth interventions range from sending simple short message service (SMS) text message reminders to attend health care appointments and downloading health-related applications for use on mobile phones, to more complex technology that records real-time patient-generated data from wearable and nonwearable sensors. Recent research has explored the potential use of mHealth interventions in improving patient health outcomes, as well as its efficacy in managing chronic conditions such as diabetes, heart disease, and cystic fibrosis [2,3].

Among mHealth interventions, evidence regarding the feasibility and acceptance of smartphones is encouraging. As portable, cheap, and convenient devices [4,5], smartphones make good candidates for the delivery of behavioral interventions [4,5]. Furthermore, they offer the opportunity to bring behavioral interventions into important real-life contexts, which facilitate decision making and self-management of patients with chronic conditions particularly [5,6]. In addition to facilitating the sharing of behavioral and clinical data with health care professionals or peers, smartphones use internal sensors to infer contexts such as user location, movement, and emotion [7,8]. This facilitates continuous and automated tracking of health-related behaviors to provide timely and tailored interventions for patients.

Despite the vast attention paid to this new field, encouraging and sustained changes in health behavior require robust evidence to understand which methods are appropriate to develop smartphone interventions and who needs to be involved in the design and development of such interventions. Recent studies have addressed the lack of evidence with respect to health care professionals’ involvement in the design and development of mHealth interventions, raising concerns regarding the reliability and accuracy of their medical content and the consequences for patient safety [9]. Since the very nature of smartphones poses a potential risk, and medical apps are increasingly used to support the diagnosis and management of diseases, facilitating health care professionals’ involvement in the developmental process is crucial. Although vital, there is little in-depth, qualitative research that allows health care providers to describe their experiences, views, and strategies in providing mHealth interventions for patients with chronic conditions [10].

One appropriate method for mHealth development and customization is conducting focus groups [11]. Focus groups have several benefits for research since the qualitative contexts provide insight into social relations, and the information obtained during the discussion reflects the social and overlapping nature of knowledge. This is more informative than a summation of individual narratives through interviews and surveys [12]. Furthermore, the focus group discussion enables researchers to collect and analyze three forms of data including individual, group level, and the data generated based on participant interaction [13]. Although it is a well-established method in qualitative research, there is a lack of guidance regarding the use of focus groups for the development of digital interventions. Focus groups are a valuable tool for identifying and dissecting the knowledge, attitudes, and perceptions that influence an individual’s behavior, as well as the barriers to and facilitators of behavioral change [14]. We used a qualitative focus group of health care professionals to identify the delivery requirements for an mHealth intervention for the postdischarge management of patients with ACS and to understand possible barriers and facilitators. To the best of our knowledge, this has not been addressed in this cohort of patients.

## Methods

### Study Aim

The present qualitative study was conducted as part of the Mobile Technology Enabled Rehabilitation-Acute Coronary Syndrome (MoTER-ACS) project. The overall aim of this project was to provide smartphone-based postdischarge educational and health interventions for patients with ACS. There were several reasons why focus group interviews were chosen as the method of data collection. First, this allowed us to identify a wide range of feelings, beliefs, and perspectives on the topic. Second, a group interview generates interaction and makes participants think about specific examples of strategies that would remain uncovered when using other methods of data collection, such as questionnaires or individual interviews. This interaction also makes it much easier to avoid suggestive or leading questions that hint at a specific strategy.

### Participants

We conducted a focus group with health care professionals to explore their unique viewpoints. All participants were recruited from the Department of Cardiology at the Prince Charles Hospital (TPCH), Queensland, Australia. The focus group (n=10) consisted of a multidisciplinary team of cardiovascular experts, including 2 nurse practitioners, 3 cardiologists, 2 research scientists, 2 clinical nurses, and 1 physiotherapist.

### Procedures

Participants were asked to provide written consent to the audiotaping of the session and completed a short questionnaire on demographics information and their professional roles in the Department of Cardiology. Subsequently, the topic of the focus group was introduced, and to facilitate an open discussion, it was emphasized that participants were free to express their opinions, with both positive and negative responses being respected. In order to help the health care professionals become familiar with the specifications of the MoTER-ACS application and its Web portal, we presented a short video clip and a PowerPoint presentation of the features of a previously developed application and its portal for cardiac rehabilitation [15]. The smartphone app and its Web portal were used for the demonstration of graphs, content, and exercises.

In order to obtain standardization and consistency, a semistructured questioning guide had been developed by the researcher, which started with an icebreaker question asking participants about their experiences of using information communications technology-based interventions during their professional practice. The questioning guide included topics relevant to the contents and strategies that health care providers are interested in when considering a smartphone-based app, and the possible barriers to the engagement of patients with the intervention. Examples of topics and related questions are presented in Table 1. The study procedure was approved by the TPCH Human Research Ethic Committee.

### Data Analysis

**Figure 1:**
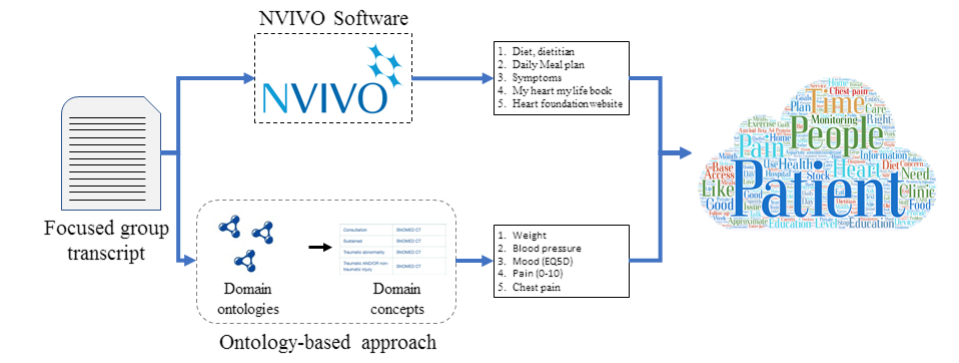
An overview of data analysis methods.

Two different methods were used for the analysis of focus group data. Figure 1 shows an overview of the study data analysis methods. The first method was an inductive content analysis that resulted in themes emerging from the data [16]. Verbatim transcriptions of the audiotaped session were generated, which then researcher read to familiarize herself with the data. The focus group transcriptions were imported into NVivo 11.0 for thematic analysis [17]. The NVivo software helps researchers to manage and organize data, facilitating the process of analysis, the identification of themes, the collection of insight, and the drawing of conclusions [18].

Thematic analysis allows the identification of themes from different levels within the data. An important part of this qualitative method of analysis was to devise a coding framework that helped to structure and reveal themes within the text [19]. We assigned codes to the text fragments, reflecting the words spoken by the participants in a more abstract way. Finally, the number of codes was reduced by combining similar codes into more comprehensive themes [16], and the expectations per questions resulted in a recommendation to include (clear majority of positive expectations) or exclude (clear majority of negative expectations) each theme in a smartphone-based intervention. NVivo requires the researcher to code the data and develop themes or categories; therefore, one can argue that the data analysis is principally subjective and allows the researcher to engage more meaningfully in the process of analysis. In order to reduce bias toward the identification of subjective themes, we investigated the impact of medical domain concepts on the analysis of the focus group data in our second approach. We employed semantic technologies, more concretely domain ontologies, which contain domain “concepts,” their definitions, and their semantic relationships to each other, to extract the medical or clinical concepts from the focus group transcriptions.

**Table 1:** Examples of the questions used in the focus group discussion.

Domain	Question
Previous experience in the use of information communication technology (ICT) interventions	Did you have experience using any ICT based intervention for patient in your clinic or ward?
Contents of a smartphone-based postdischarge intervention for patients with acute coronary syndrome	In your opinion, what are the needs of patient with acute coronary syndrome that can be addressed via mobile phone based clinic? What don’t you like to consider in the mobile phone based multidisciplinary clinic?
Concerns regarding a smartphone-based postdischarge intervention for patients with acute coronary syndrome	In your opinion, what are some concerns about the mobile phone–based clinic?

**Table 2:** Summary of selected ontologies.

Ontology	Description	Selection criterion
SNOMED CT	Systematized Nomenclature of Medicine - Clinical Terms	Quantitative
LOINC	Logical Observation Identifier Names and Codes	Quantitative
MESH	Medical Subject Headings	Quantitative
NCIT	National Cancer Institute Thesaurus (a vocabulary for clinical care, translational and basic research, and public information and administrative activities)	Quantitative
RCD	Read Codes, Clinical Terms Version 3	Quantitative
NIC	Nursing Interventions Classification	Qualitative
ICNP	International Classification for Nursing Practice	Qualitative
NCCO	Nursing Care Coordination Ontology (contains activities in which nurses engage while coordinating care among patients)	Qualitative
APAONTO	Psychology Ontology	Qualitative
ONTOPSYCHIA	OntoPsychia, social module (ontology of social and environmental determinants for psychiatry)	Qualitative

Due to their narrative nature, the transcriptions of the focus groups usually exhibit noisy and inconsistent characteristics in terms of the terminologies used throughout the discussion (eg, “heart attack” disorder may be referred to as “cardiac infarction,” “myocardial infarction,” or simply “MI” by different participants in the focus group). The main advantage of considering concepts in such analyses compared with a word-level analysis is that by normalizing the transcriptions to their concepts, semantically similar terms and phrases are consolidated and a large portion of unrelated terms are removed. Hence, this concept-level representation of the transcripts can provide a solid platform for more effective pinpointing of the essential themes. Our concept-level analysis was performed through the following steps: (1) selecting the appropriate ontology, (2) annotating the transcripts using the selected ontologies, and (3) analyzing the extracted concepts and mapping or deriving codes or themes from them. The details of these steps are described below.

#### Ontology Selection

We selected the most appropriate ontologies based on 2 different quantitative and qualitative criteria. In the former method, the ontologies were selected according to an ontology recommender system [20] that is available from National Center for Biomedical Ontology (NCBO) BioPortal [21]. The NCBO BioPortal [21] is a comprehensive repository of ontologies, which hosts over 500 different ontologies in the biomedical domain [22]. For a given dataset or corpus, the NCBO ontology recommender system ranks the suitability of the ontologies according to their coverage (ie, the more the concepts from an ontology appear in the given corpus, the higher their final ranking). As shown in Table 2, we reviewed the top 5 ontologies for each question in the focus group that were suggested by the ontology recommender system, finally selecting the 5 most frequent ontologies among the questions. From the qualitative perspective, we searched and reviewed medical ontologies and their descriptions to find more related ontologies in terms of their relatedness to the core disciplines to which our focus group belong (ie, mHealth, nursing, and psychology). In this approach, we retrieved and analyzed the available ontologies in the NCBO BioPortal, which led us to select 3 ontologies in the nursing domain and 2 ontologies in the realm of psychology (to the best of our knowledge, there is no available mHealth-related ontology in the NCBO BioPortal).

#### Annotation

The next step after ontology selection was the annotation process (ie, locating spans of text in the transcripts that refer to the concepts defined in the ontologies). In order to annotate the collected answers to the questions within the focus group data, we employed the NCBO concept annotator [23]. We developed a Java program that submits the text to the annotator through a RESTful API, a technology to communicate with Web services or applications, and processes the returned annotations (in an XML format) to extract the annotated concepts [24].

#### Concept Analysis and Code Derivation

The extracted concepts from all ontologies were combined, followed by calculation of their frequencies, both at the question level and the full transcript level. The analysis of the more frequent concepts led us to derive a number of new themes and to support the identified codes chosen during the manual analysis of the transcripts in the above-mentioned method.

## Results

### Main Findings

In total, 10 health care providers from the Department of Cardiology took part in this study. Among these, 70% (7/10) had experience in using mHealth interventions in their clinic or ward. Participants’ characteristics are depicted in Table 3, demonstrating the diversity of health care providers with a range of disciplines and backgrounds. The study results are presented as major themes in Table 4. We presented recommendations for a mobile phone–based app and its Web portal to provide health interventions to patients with ACS.

### Educational Instructions

The first topic that participants recommended for consideration with respect to patients with ACS was educational materials, including instructions on diet, providing daily meal plans, and education on ACS symptoms and its concepts, considering the patient’s condition. Participants recommended to use the Heart Foundation website and the “My heart, my life” book as resources for patient education [25].

More education can balance the symptoms to qualify chest pain.

Education of ACS symptoms is necessary.

From my point of view as physio, education and exercise are essential for our patients.

When we educate the patients we introduce them the ’my heart my life’ book published by the Heart Foundation. And when the time comes, they know couple of meal options that they like and have an idea about the portion sizes that they can and can’t eat. All the patients find it very helpful.

That’s why they are very keen on the information they can get, they do like a meal plan.

### Health Measures

Four items were identified by the health care providers to be measured via the smartphone-based app, including body weight, blood pressure, mood, and pain. The use of the tool “European Quality of life Questionnaire-five dimensions” [26] was recommended to assess patients’ health status. Health care providers recommended assessing the pain level of patients with ACS using a scale of 0-10.

Yes that’s EQ5D, 10 points is great for mood assessment.

### Not Recommended for Self-Monitoring

Two ACS symptoms, shortness of breath and chest pain, were identified as items that health care professionals provided a negative opinion regarding daily monitoring via the smartphone app. Furthermore, participants indicated that assessing patients’ electrocardiographs is not useful.

Shortness of breathing is the biggest issue to measure through phone apps. When I have started my ACS clinic, on the first 6 months, I see all the patients have chest pain. Then you start to work out how to differentiate that type of shortness of breath vs sleep apnoea vs asthma vs life time smoker, COPD type disease. I think if we put generalised options such as pre-chest pain, chest tightness, shortness of breath, we would get this very long by some patients as they are very good at writing in.

You can’t put that [the options in app that asks about patient’s chest pain], because I think they would actually record chest pains. For example, if I press here I can get a sort of chest pain right now.

How do we then have to react to that, can we just say it doesn’t mean anything?

**Table 3:** Participant characteristics.

Characteristics		Value
Age in years, mean (SD)		47 (8)
**Gender, n (%)**		
	Male	5 (50)
	Female	5 (50)
**Marital status, n (%)**		
	Widowed or divorced	0 (0)
	Married	8 (80)
	De facto or other	1 (10)
	Single	1 (10)
**Highest level of education, n (%)**		
	<12 years	0 (0)
	High school diploma	1 (10)
	Some college or associates degree	0 (0)
	Postgraduate degree	9 (90)
**Profession, n (%)**		
	Cardiologist	3 (30)
	Nurse practitioner	2 (20)
	Research scientist	2 (20)
	Clinical nurse	2 (20)
	Physiotherapist	1 (10)

**Table 4:** Major themes.

Major themes and subthemes		References extracted from the transcription, n (%)
**Educational instructions**		
	Diet, dietitian	11 (10.6)
	Daily meal plan	7 (6.7)
	Symptoms (define concepts)	7 (6.7)
	“My heart, my life” book	4 (3.8)
	Heart Foundation website	5 (4.8)
**Health measures**		
	Weight	4 (3.8)
	Blood pressure	4 (3.8)
	Mood (European Quality of Life Questionnaire-five dimensions)	8 (7.7)
	Pain (0-10)	6 (5.8)
**Not recommended for self-monitoring**		
	Electrocardiograph	3 (2.9)
	Chest pain	5 (4.8)
	Shortness of breath	5 (4.8)
**Real-time communication**		
	Nonverbal	3 (2.9)
	FaceTime	4 (3.8)
	Video calling	4 (3.8)
**Engagement or motivational barriers**		
	Older age	5 (4.8)
	Educational level	2 (1.9)
	Access to technology	2 (1.9)
	Staff workload	3 (2.9)
**Monitoring or alarm**		
	Monitoring mechanism	3 (2.9)
	Contacting patient when alarm is off	4 (3.8)
**Intervention follow-up**		
	Long-term vs short-term	4 (3.8)

### Real-Time Communication

Another strategy mentioned by the focus group participants was providing a communication facility for health care providers with which to communicate with patients when required. Video conferencing applications (eg, FaceTime) were identified as useful tools that facilitate communication between health care providers and patients. Nonverbal communication and observation of patients’ body language through videoconferencing were also identified as useful assessment tools.

Ideally daily monitoring; but you can’t see a patient in clinic every day. Realistically, you see a patient in a month or few months in clinic.

Because there is so much nonverbal guide and I hate communication through phone because you don’t get the non-verbals.

Adding a FaceTime-equivalent option to phone calls is great. Because health related video calls might be more useful than just phone calls.

### Engagement or Motivational Barriers

Older age, low educational level, and lack of access to technology were identified as patient barriers to engage with smartphone-based interventions. Participants also mentioned that staff workload may increase due to monitoring and providing online support and this would be considered as a potential barrier from health care providers’ perspective.

Younger population shouldn’t be a problem, but, there are concerns about older population.

They have to have internet access. They have to have mobile phone and they have to have an educational level where they have ability to read and understand.

Such phone-based intervention is great and will improve patient care but it can also add lots of workload on us through increase of online patients communications. In a classic way, we only see patients at clinic and then they go home but with this we need to follow-up.

### Monitoring Mechanism or Alarm

Health care providers pointed out using a monitoring mechanism or alarm system within the portal to inform clinicians of patients’ daily health measures and intervention usage.

I think you should identify what you just said when an alert goes off something needs to be done so people don’t start dropping down with their exercise and if there’s a red flag someone has to call them and get them to do what we want and see’s what happens if they don’t.

There should be a monitoring mechanism in place when an intervention is implemented and we should find out for how long monitoring is required.

### Intervention Follow-Up

Participants acknowledged the importance of long-term follow-up versus short-term to achieve sustainability of the smartphone-based intervention for postdischarge patients.

Well, the advantages of phone-based intervention become apparent from a long-term perspective rather than a short-term follow-up period. Because, just fall back into their old habits after a while.

We need to implement it in a way to be able to follow them through a 6 and then 12 months periods. We get patients’ initial compliance and then gradually decrease their involvement over time period.

## Discussion

### Principal Findings

In this qualitative study, we investigated the expectations of health care professionals with respect to a smartphone-based app and its portal to empower patients with ACS. The focus group resulted in useful feedback regarding different contents and features of such an application to provide postdischarge support and health management for patients with ACS. The important themes that emerged were educational instructions, health measures (body weight, blood pressure, mood, and pain), not recommended for self-monitoring (chest pain, shortness of breath), real-time communication, engagement and motivational barriers, monitoring or alarm mechanism, and intervention follow-up.

Health care professionals were most positive about providing educational instructions related to diet and ACS symptoms. Providing patients with daily meal plans and information related to healthy eating were strongly recommended by the participants. This is consistent with the current evidence showing that healthy eating is associated with a lower mortality risk in a large cohort of cardiac patients [27].

The most negative feedback received from the health care professionals was about the daily monitoring of ACS signs and symptoms, including chest pain and shortness of breath, via the mobile phone app. Participants pointed out that these health measures are subjective and vague and that it is required for patients to adequately understand symptoms associated with their condition and recognize the possible underlying reasons.

The health care providers suggested that measuring patient health status, including emotions and pain, would be informative in providing sustainable postdischarge support for patients with ACS. It is evidenced that depression is common after a coronary event [28], and it continues to remain underrecognized and poorly treated in the cardiac population [29]. Previous research has shown that patients with ACS benefit from cognitive behavioral therapy following an episode of myocardial infarction [30]; therefore, measuring emotional status will assist clinicians to identify depressive symptoms.

Based on our focus group results, a visual communication tool is required to assess patient health status and to provide psychosocial support and encouragement during the intervention. Studies in the fields of chronic disease and rheumatology have also found that patients considerably value face-to-face supervision by a health care professional [31,32]. Furthermore, it is known that multiple communications with clinicians result in a lower dropout rate and better adherence to interventions [33]. Accordingly, this will increase patient motivation and engagement and improve their empowerment.

Being older, having a low educational level, and lacking computer skills were identified as engagement and motivational barriers. This is consistent with the current evidence describing potential barriers for mHealth interventions. While mHealth technologies have the potential to improve population health outcomes and the delivery of health care services, there is a need to use and develop mHealth applications with caution. Using smartphone-based SMS text messaging requires a certain level of literacy. In addition, researching the use of “apps” to provide education and patient engagement in elderly populations may be hindered by the prevalence or access of certain technologies, such as smartphones, within this population. While the mobile platform remains flexible to engage patients via written, verbal, or video interactions, there is a need to consider how the elderly or individuals without advanced technical skills will interact with the device or participate in the intervention [34].

### Strengths and Limitations

The strengths of the study design include providing structured questions during the focus group, as well as screenshots of the smartphone app. Furthermore, the themes that emerged from the thematic analysis were validated using concept mapping methodology. Facilitators were successful in creating a comfortable conversation environment, and participants felt confident in raising their positive and negative opinions on smartphone interventions for the postdischarge management of patients with ACS. A few limitations should be considered. Due to time restrictions, we did not ask participants to discuss application usability or appearance; hence, this will be investigated in a future study with patient participants. Although clinicians are one of the key stakeholders in the use of mHealth technologies, the development of such interventions requires an iterative process of obtaining information and guidance from all stakeholders, including patients, information technology specialists, and providers. In this study, we aimed to focus on clinician perspectives, and patient perspectives will be investigated in a future study using different methodologies.

## Abbreviations

ACS: acute coronary syndrome
eHealth: electronic health
mHealth: mobile health
MoTER-ACS: Mobile Technology Enabled Rehabilitation-Acute Coronary Syndrome
NCBO: National Center for Biomedical Ontology
SMS: short message service
TPCH: The Prince Charles Hospital
